# Incorporating digitally derived endpoints within clinical development programs by leveraging prior work

**DOI:** 10.1038/s41746-023-00886-9

**Published:** 2023-08-10

**Authors:** Amy Bertha, Rinol Alaj, Imein Bousnina, Megan K. Doyle, Danielle Friend, Rasika Kalamegham, Lauren Oliva, Igor Knezevic, Frank Kramer, Hans-Peter Podhaisky, Sven Reimann

**Affiliations:** 1 Bayer, 801 Pennsylvania Ave NW, Washington, DC 20004 USA; 2 Regeneron, Terrytown, NJ USA; 3 Genentech, South San Francisco, CA USA; 4 Amgen, Thousand Oaks, CA USA; 5 Janssen, Raritan, NJ USA; 6 Biogen, Cambridge, MA USA; 7 Bayer, Wuppertal, Germany; 8 Bayer, Berlin, Germany

**Keywords:** Biomarkers, Policy

## Abstract

Digital health technologies (DHTs) enable remote data collection, support a patient-centric approach to drug development, and provide real-time data in real-world settings. With increasing use of DHTs in clinical care and development, we expect a growing body of evidence supporting use of DHTs to capture endpoint data in clinical trials. As the body of evidence grows, it will be critical to ensure that available prior work can be leveraged. We propose a framework to reuse analytical and clinical validation, as well as verification data, generated for existing DHTs. We apply real life case studies to illustrate our proposal aimed at leveraging prior work, while applying the V3 framework (verification, analytical validation, clinical validation) and avoiding duplication. Utilizing our framework will enable stakeholders to share best practices and consistent approaches to employing these tools in clinical studies, build on each other’s work, and ultimately accelerate evidence generation demonstrating the reproducibility and value add of these new tools.

## Introduction

The use of digital health technologies (DHTs) and digitally derived endpoints are part of modern, innovative clinical development programs (CDPs). A DHT is a system that uses computing platforms, connectivity, software, and/or sensors for healthcare and related uses^1^. DHTs support patient-centric approaches and decentralized clinical trial activities (we use the term DHT, as it is preferred by regulatory health authorities). DHTs can objectively measure clinically meaningful aspects of health that have been difficult or impossible to measure previously in a real-world setting and in a continuous manner, as opposed to point-in-time assessments. Clinical trials utilizing DHTs have the potential to engage more patients by enhancing trial participation for geographically remote participants and participants who face difficulties traveling to clinical trial sites and by reducing burden on trial participants through remote data collection.

The V3 framework^2^ describes a three-stage process of Verifying, analytically Validating, and clinically Validating a Biometric Monitoring Tool (BioMeT) to demonstrate it is fit-for-purpose for gathering data in a clinical trial. Since its introduction, many new DHTs, which includes BioMeTs, have been developed and deployed. As sponsors began to gain experience with their use in CDPs, they recognized the need for additional clarity and many authors have sought to address this need^3–12^. In actuality multiple entities, such as DHT developers, sponsors, and other stakeholders, are performing the various aspects of V3 across multiple medical product development programs. Thus, there was an opportunity to leverage available V3 work without requiring additional studies for each new CDP. In 2021, U.S. Food and Drug Administration (FDA) draft guidance *Digital Health Technologies for Remote Data Acquisition in Clinical Investigations*^1^also stated that prior work could be leveraged, but did not provide a framework to do so.

We propose a framework that addresses how sponsors of clinical trials can implement the V3 approach. We outline key considerations for sponsors incorporating DHTs and digitally derived endpoints within their CDPs. The framework addresses the regulatory status of the DHT and what information might be leveraged to support a clinical trial application. We have highlighted the most common instances of DHT use in clinical trials and designed our framework to be intentionally broad to allow for adaptations, as additional uses become apparent. Finally, we include a case example of leveraging prior work.

## Discussion

### Incorporating a DHT and digitally derived endpoint within a CDP

The work to incorporate a digitally derived endpoint into clinical development and ensure the DHT is accurate and reliable for regulatory decision making could take several years and needs to be factored into timelines. Deliberations on incorporating a digitally derived endpoint should begin as early as possible and before a clinical protocol is written. As a general matter, close collaboration between the medical product developer and DHT developer (if separate entities) is maintained throughout the development program. We recommend early, continuous, and close communication with regulatory health authorities. As a best practice, in the U.S. context, sponsors developing human medical products should consider requesting formal meetings^13^ with FDA early and often to discuss the use of a DHT and digitally derived endpoint within the context of a specific clinical development program. For more broad discussions on the use of a DHT outside a specific clinical development program, requesting a FDA Critical Path Innovation Meeting^14^, qualifying a tool for a specific context-of-use through FDA’s Drug Development Tool qualification program^15^, or participating in FDA’s Innovation Science and Technology Approaches for New Drugs (ISTAND) pilot program^16^ may be more appropriate.

Sponsors of clinical trials intending to incorporate DHTs should start while in the discovery/preclinical phase of development programs. Key factors to consider during this phase include, conducting literature review, researching the technology landscape, and establishing the concept of interest and context of use^17^. Once the need for a DHT is established, sponsors should develop a validation plan and conduct verification of a DHT in healthy volunteers, if appropriate. At this point, sponsors may consider including discussions of the conceptual framework, including the digitally derived endpoint development plan according to the V3 framework, with FDA in a Type B pre-investigational new drug application (pre-IND) meeting or a Type C meeting, if First-in-Human trials have already started.

During Phase 1, sponsors should select the DHT, conduct a gap assessment of the existing verification and validation data (Figs. 2 and 3), and consider whether to initiate a pilot study of the DHT in the target patient population. A sponsor may not be in a position to know what the DHT can measure until it is tested in a sample of the intent-to-treat (ITT) population. One way to test in the ITT population is to perform a Phase 1 study using the digitally derived endpoint in an exploratory fashion. At this point, sponsors may consider discussing the specific DHT selected and the plans for the exploratory use of the digitally derived endpoint in a Type C meeting. This discussion may include the outcome of the gap assessment, including what prior work is intended to be leveraged to support verification and validation and what additional work is intended to be performed.

During Phase 2, the sponsor should test usability of the DHT, as well as validate the exploratory digitally derived endpoint. During Phase 3, the selected DHT and digitally derived endpoint can be employed in the registrational studies. The use of the DHT and digitally derived endpoint can be further refined during Phase 4, as appropriate. It is critical that sponsors gain FDA acceptance of the final verification, analytical validation and clinical validation package and digitally derived endpoint before starting the pivotal phase 3 trial. This discussion may be included, for example, in a Type B end-of-phase 2 meeting.

**Fig. 1:**
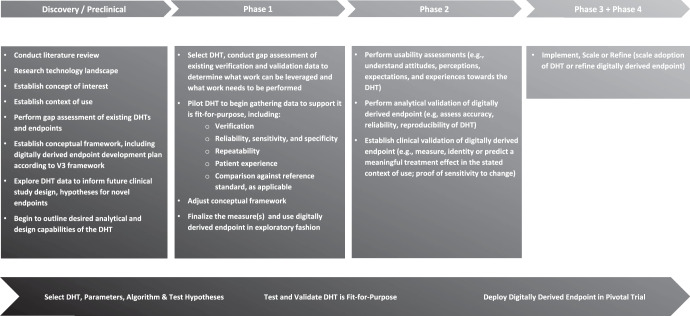
A strategic approach to incorporating a digital health technology and digitally derived endpoint within a clinical development program. To demonstrate that the digital health technology (DHT) is fit-for-purpose in regulatory decision making, all of the steps here should be completed, but the order of execution and when they are completed, in terms of drug development phases, may vary. Certain drug development programs may not follow the precise three-phase structure shown here, and some activities shown here may be completed in parallel with one another. In a regulatory context, the concept of interest is the aspect of an individual’s clinical, biological, physical, or functional state, or experience that the assessment is intended to capture (or reflect). Context of Use is a statement that fully and clearly describes the way the medical product development tool is to be used and the regulated product development and review-related purpose of the use. V3 framework^2^ refers to a three-stage process of Verifying, analytically Validating, and clinically Validating a Biometric Monitoring Tool (BioMeT) to demonstrate it is fit-for-purpose for gathering data in a clinical trial.

Figure 1 outlines activities that could be conducted in parallel or at different timepoints. Further, the activities are not prescriptive, but rather illustrate how DHT and digitally derived endpoint development could map to the medical product development process.

### Leveraging prior work to demonstrate a DHT and digitally derived endpoint are fit-for-purpose

As the use of DHTs expands, the pharmaceutical industry and other stakeholders will have access to a growing body of evidence supporting their use to capture endpoints. It is important that data generated by the various stakeholders can be utilized for maximal benefit of patients by ensuring prior work can be leveraged. We envision prior work to include data that is publicly available or for which the manufacturer has a right of reference, e.g., published data, regulatory status, datasets, concept of interest, and usability, as well as work generated to support use of the endpoint, that are legally available to the sponsor. It follows that the extent and quality of prior work will vary depending on whether the tool was reviewed by the regulatory health authority and the specific medical device regulatory pathway used, i.e. 510(k) clearance, Pre-Market Application, CE Mark. Although an important consideration, we will not be differentiating what type of prior work can or should be able to be leveraged depending on which medical device regulatory pathway was used.

Sponsors need to estimate clinical development costs and timelines to adequately resource development programs. Hence, sponsors need clarity about the level of evidence that must be generated, how it can be generated, and the timeline for gathering the evidence. Allowing prior work to be leveraged can help build the evidentiary base for the use of a specific DHT in a clinical study, provide data or confirm the validity of a digitally derived endpoint, and allow sponsors to estimate risk and resources needed to successfully employ DHTs in clinical programs. This information is critical to make informed decisions and ensure successful execution of CDPs.

**Fig. 2:**
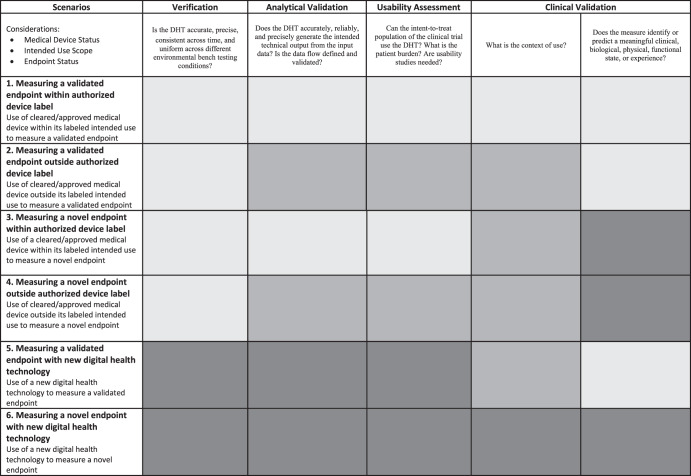
A Framework for leveraging prior work to demonstrate a digital health technology (DHT) and digitally derived endpoint are fit-for-purpose—an overview. Prior Work can and should be leveraged in all scenarios where available and appropriate, shading indicates when additional work may or likely will be needed. The light gray shading indicates no additional work is needed. Sponsors can leverage prior work for all aspects of verification, validation, and usability. The medium gray shading indicates additional work may be needed. Sponsors will need to confirm what work can be leveraged, determine if additional work is needed, and perform needed work to support certain activities. The dark gray shading indicates additional work likely is needed. Sponsors will need to generate most data de novo.

**Fig. 3:**
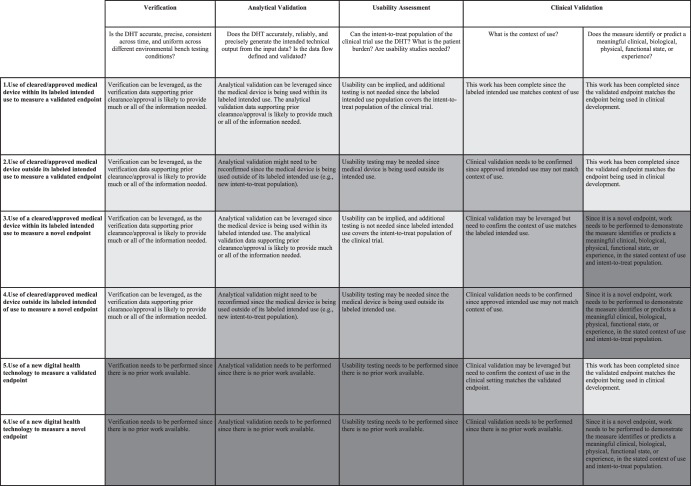
A framework for leveraging prior work to demonstrate a digital health technology (DHT) and digitally derived endpoint is fit-for-purpose—the detail. Prior Work can and should be leveraged in all scenarios where available and appropriate, shading indicates when additional work may or likely will be needed. The light gray shading indicates no additional work is needed. The medium gray shading indicates additional work may be needed. The dark gray shading indicates additional work likely is needed.

Figure 1 represents an idealized drug development scenario. We propose a framework in Fig. 2 that illustrates how to employ DHTs in various scenarios recognizing that drug development and DHT validation are not always synchronous, and prior work may exist that could be leveraged. The framework outlines a methodology based on applying the V3 paradigm, leveraging prior work, and conducting appropriate gap assessments, to determine what additional evidence should be generated to support use of a DHT in individual CDPs. The framework can facilitate planning for a trial employing a DHT with an emphasis on the verification and analytic validation of the DHT. The framework encourages leveraging prior work, to ensure robust verification and validation while avoiding duplication. Figure 2 outlines the consolidated framework. Figure 3 provides more details. The sponsor’s assessment of the data and plan to leverage prior work, including rationale, will need to be endorsed by regulatory health authorities.

### Case study of leveraging prior work

FDA’s draft guidance *Digital Health Technologies for Remote Data Acquisition in Clinical Investigations: Draft Guidance for Industry, Investigators, and Other Stakeholders*, conveniently provides several case study examples^1^. Using one such example we will illustrate the applicability and utility of our framework. A sponsor is considering using a portable wearable device in a clinical investigation of a new drug for the treatment of insomnia disorder. The DHT under consideration has already received FDA marketing authorization to remotely measure sleep parameters in the home setting. The current methods for assessing sleep parameters in clinical investigations are based on diary-recorded participant estimates or on polysomnography (PSG) conducted in a sleep laboratory.

Based on the Framework for Leveraging Prior Work (Fig. 2), this example best fits Scenario 3: Measuring a novel endpoint within an authorized label. The rationale for this choice is (1) the DHT has previously received FDA marketing authorization; (2) use of the DHT is within the intended use of the marketing authorization (to remotely measure sleep parameters in the home setting); and (3) use of the DHT allows for increased monitoring frequency which presents opportunities to construct novel endpoints that rely on multiple data points. Scenario 3 of the Framework outlines that prior work may likely be leveraged for verification, analytical validation, and usability evaluations; however, additional work will likely be necessary to clinically validate the DHT for use in the clinical investigation. Prior work in this context refers to analyses completed during the development of the DHT as a medical device, including verification of sensor data, analytical and clinical validation and usability assessments. In addition, in accordance with the V3 framework, we consider the analytical validation work performed in the context of the medical device review in healthy volunteers to still apply in the context of insomnia patients as the concept of interest studied in the drug trial can be assessed in healthy volunteers. Indeed, we believe that since the DHT is to be used within the same intended use of the marketing authorization, the data that satisfied device regulators’ requirements should also help address the drug regulators’ evidence need.

In this example, the DHT is designed to measure the same sleep parameters in the same setting as the intended use of the authorized device. It is important to note that although the concept of interest (i.e., sleep parameters for insomnia disorder) is not novel for regulatory purposes (i.e., similar to the data captured by existing diary-recorded and PSG methods), the endpoint may still be considered novel necessitating the sponsor to demonstrate that the portable wearable device data is equivalent or superior to the data collected by the current method. The sponsor may also be able to leverage prior work on the clinical meaningfulness of select sleep parameters for patients with insomnia disorder to support use of the DHT in the clinical investigation.

**Fig. 4:**
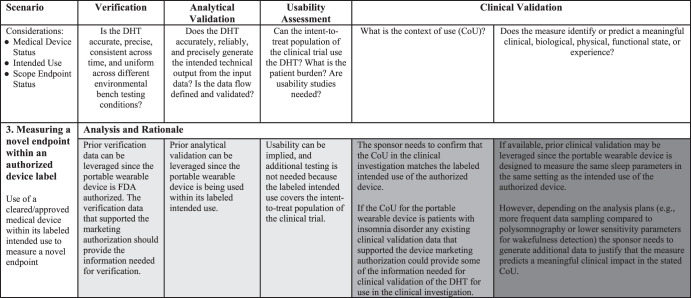
A framework for leveraging prior work to demonstrate a digital health technology (DHT) and digitally derived endpoint is fit-for-purpose—case study in remote monitoring sleep parameter in home setting. Prior Work can and should be leveraged in all scenarios where available and appropriate, shading indicates when additional work may or likely will be needed. The light gray shading indicates no additional work is needed. The medium gray shading indicates additional work may be needed. The dark gray shading indicates additional work likely is needed.

In Fig. 4, we outline detailed considerations for the sponsors in this example to determine if prior data may be leveraged to demonstrate that the DHT is fit-for-purpose. As already noted, Fig. 2 is a general guideline, and re-use of verification and validation data will vary depending on the specifics of the DHT, the measure, and prior regulatory experience.

## Conclusion

We outline a strategic approach to incorporating a DHT and digitally derived endpoint within a CDP and elements to consider during each phase of implementation. We have also built upon the V3 framework concept and developed practical considerations to employ a DHT in a clinical investigation. Our approach will help trial sponsors assess the viability of using a DHT in a CDP; assess evidentiary gaps; and, simultaneously leverage existing data efficiently.

We believe our approach is consistent with recent health authority guidance on the topic which underlines the importance of collective data to demonstrate new tools, technologies, and methodologies are fit-for-purpose. Thus, effective multi-stakeholder collaboration will lead to efficient and timely deployment of DHTs. We recommend early and close communication with regulatory health authorities to ensure the validation plan will address their evidentiary needs. While this paper is focused on verification and validation of the DHT, it should be noted that validation of the measure itself will also be needed but is out of scope for the current work^18^.

Leveraging prior work is critical to enable stakeholders to share best practices and consistent approaches to employing DHTs in clinical studies, build on each other’s work, and collaborate to generate evidence demonstrating the reproducibility and value of DHTs. We have created a living framework that sponsors may use to assess how to leverage existing evidence to demonstrate that a DHT is fit-for-purpose in an individual CDP, while maintaining high evidentiary standards. We hope sponsors will use this framework in practice and the stakeholder community will build upon this work.

## Data Availability

Data sharing is not applicable to this article as no datasets were generated or analyzed in relation to this article.

## References

[1] U.S. Food and Drug Administration. Draft Guidance for Industry: Investigators, and Other Stakeholders. Digital Health Technologies for Remote Data Acquisition in Clinical Investigations (2021) Guidance for Industry (fda.gov).

[2] Goldsack J (2020). Verification, analytical validation, and clinical validation (V3): the foundation of determining fit-for-purpose for Biometric Monitoring Technologies (BioMeTs). npj Digit. Med..

[3] Crouthamel M (2021). Developing a novel measurement of sleep in rheumatoid arthritis: study proposal for approach and considerations. Digit Biomark..

[4] Izmailova E (2019). Evaluation of wearable digital devices in a phase I clinical trial. Clin. Transl. Sci..

[5] Godfrey A (2021). Fit-for-purpose biometric monitoring technologies: leveraging the laboratory biomarker experience. Clin. Transl. Sci..

[6] Coravos A (2019). Digital medicine: a primer on measurement. Digit Biomark..

[7] Coravos A (2020). Modernizing and designing evaluation frameworks for connected sensor technologies in medicine. npj Digit. Med..

[8] Li Y (2019). Use digital sensor and deep learning to evaluate motor performance in the D1PAM (LY3154207) phase 1B Parkinson’s disease clinical trial [abstract]. Mov Disord..

[9] Stephenson D (2020). Precompetitive consensus building to facilitate the use of digital health technologies to support parkinson disease drug development through regulatory science. Digit Biomark..

[10] Colloud S (2023). Evolving regulatory perspectives on digital health technologies for medicinal product development. npj Digit. Med..

[11] Gelis L, Stoeckert I, Podhaisky H-P (2023). Digital tools – regulatory considerations for application in clinical trials. Ther. Innov. Regul. Sci..

[12] Perry B (2023). How much evidence is enough? research sponsor experiences seeking regulatory acceptance of digital health technology-derived endpoints. Digit Biomark..

[13] U.S Food and Drug Administration. Guidance for Industry: Formal Meetings Between the FDA and Sponsors or Applicants of PDUFA Products (2017).

[14] U.S. Food and Drug Administration. Guidance for Industry: Critical Path Innovation Meetings (2015) Critical Path Innovation Meetings Guidance for Industry (fda.gov).

[15] U.S. Food and Drug Administration. Guidance for Industry and FDA Staff: Qualification Process for Drug Development Tools (2020) Guidance for Industry (fda.gov).

[16] U.S. Food and Drug Administration. Innovation Science and Technology Approaches for New Drugs (ISTAND) Pilot Program [Internet] (2023).

[17] FDA-NIH Biomarker Working Group. BEST (Biomarkers, EndpointS, and other Tools) Resource [Internet] Silver Spring (MD) 2016. 27010052

[18] U.S. Food and Drug Administration. Draft Guidance for Industry, Food and Drug Administration Staff, and Other Stakeholders: Patient-Focused Drug Development: Selecting, Developing, or Modifying Fit-for Purpose Clinical Outcome Assessments (2022).

